# Improving Acute Resuscitation Plan Completion Rates Using Visual Aids in an Inpatient Setting: A Quality Improvement Study

**DOI:** 10.7759/cureus.118318

**Published:** 2026-10-21

**Authors:** Michael T Byrne, Adam G Selway

**Affiliations:** 1 Internal Medicine Department, Logan Hospital, Loganlea, AUS; 2 Medicine Department, The University of Queensland, Brisbane, AUS; 3 Emergency Department, Royal Brisbane and Women's Hospital, Brisbane, AUS

**Keywords:** acute resuscitation plans, patient safety, quality improvement, resuscitation planning, visual aids

## Abstract

Background

An Acute Resuscitation Plan (ARP) is a document that helps medical professionals incorporate the wishes of patients and their families, as well as the opinions of the treating team, when making decisions about treatment escalation and whether resuscitation is appropriate should a patient clinically deteriorate. This project aimed to assess if the use of visual aids increased the completion rate of ARPs in an inpatient cardiology setting.

Methods

We completed a two-cycle quality improvement study using the plan-do-study-act framework, starting with an initial audit before introducing interventions in the form of visual aids over three weeks. Re-auditing occurred each week to assess the impact of the interventions on inpatient ARP completion.

Results

The baseline audit, conducted prior to any interventions, showed an ARP completion rate of 16% across all patients admitted under cardiology. After the first cycle of the project involving the introduction of posters and a change to the cardiology doctor’s ward list, the ARP completion rate increased to 47.6% (p = 0.027). After a second cycle, introducing further visual aids attached to mobile workstations, the ARP completion rate rose to 52.2% (p = 0.014), showing a significant rise compared to baseline.

Conclusions

This project demonstrated that the use of visual aids significantly increased the rate of ARP completion in an inpatient cardiology setting. To further assess the success of these interventions, we suggest expanding this project hospital-wide and monitoring the ARP completion rate over a prolonged period to demonstrate the effectiveness of these interventions in the longer term.

## Introduction

An Acute Resuscitation Plan (ARP) is a document to help medical professionals incorporate the wishes of patients and their families, as well as the opinion of the treating team, when making decisions about escalation of treatment and whether resuscitation is appropriate, should a patient clinically deteriorate [1,2]. It stipulates what treatments are appropriate to provide in the context of deterioration, and those that are not appropriate, including whether cardiopulmonary resuscitation (CPR) should be provided [1,2]. Patients deemed not suitable for CPR should have a do not attempt CPR (DNACPR) order as part of their ARP [1,2].

ARPs have previously been shown to reduce the number of patients inappropriately undergoing resuscitation and have been effective at facilitating care which best reflects patients’ wishes [3,4]. Despite this, completion rates of ARPs often remain poor in inpatient settings [3,5].

There are several factors that can affect the rate of ARP completion. Patients admitted with an altered mental status, who are clinically unwell, or who have family members with conflicting views on whether resuscitation is appropriate, can cause challenges in completing ARPs [6,7]. Patients may also ask for time to fully contemplate their thoughts or to have discussions with their family, which can also delay ARP completion [4].

Clinician factors play a role too; perceived lack of physician time, a lack of confidence from junior colleagues, and the need for consultant input have previously been cited as reasons for delaying ARP completion [8].

This quality improvement (QI) project took place in a district general hospital cardiology unit consisting of an eight-bed coronary care unit (CCU) and a 32-bed ward, shared between cardiology and renal medicine, over the course of three weeks. For the purposes of this project, only patients admitted under cardiology were included in the results. Patients admitted under cardiology who were admitted to a ward other than the base cardiology ward were also included.

Our objective was to increase ARP completion rates across the department of cardiology. We hypothesised that the use of visual aids in clinical spaces would prompt a greater number of ARP discussions. In this project, visual aids were broadly defined as pictures or writing that conveyed information to clinicians.

## Materials and methods

This project utilised the plan, do, study, act methodology [9] for QI with the aim of improving the completion rates of ARPs over two cycles of interventions.

Measures

The hospital in which this project took place has an electronic system for documenting ARPs on the inpatient electronic medical record (IEMR). Patients who have a completed ARP have a tab on their patient profile indicating whether the ARP is active and its contents. It also records the date that the ARP discussion occurred. The ARP can be made active for a maximum period of one year or for the current hospital admission only.

ARP status was assessed by reviewing the ARP tab on the IEMR system for each patient admitted under the Department of Cardiology. Patients were considered to have a completed ARP if they had an active ARP for that admission. Those with previously completed but expired ARPs were not counted as having an ARP for the purposes of the study.

Baseline data collection

We performed an initial audit to assess the completion rates of ARPs in the cardiology department before any interventions were introduced.

Interventions

Interventions were implemented at the start of each working week on a Monday, and re-auditing was undertaken each Friday, allowing time for each intervention to take effect. This continued for two cycles.

The first interventions included the addition of a visual prompt to the cardiology doctor’s ward list. The ward list was an existing document that listed the current cardiology inpatients, which was printed each day and used by members of the cardiology team on ward rounds. We added a column that reflected the ARP status of each patient and whether a DNACPR was in place. Patients who did not yet have an active ARP had a blank space in this column, highlighting that they may require an ARP discussion. This ward list was available online in addition to the physical copies and could be edited by all clinicians in the cardiology team. The ARP column was not a mandatory field and did not change the process of how clinical handovers were conducted. This therefore was considered a visual prompt and did not constitute a process change. Posters were also created and placed at the workstations of clinicians working in the cardiology department, encouraging the completion of ARPs for cardiology inpatients and prompting discussion with patients and their families. An example of one of the posters used in the study can be seen in Figure 1.

**Figure 1:**
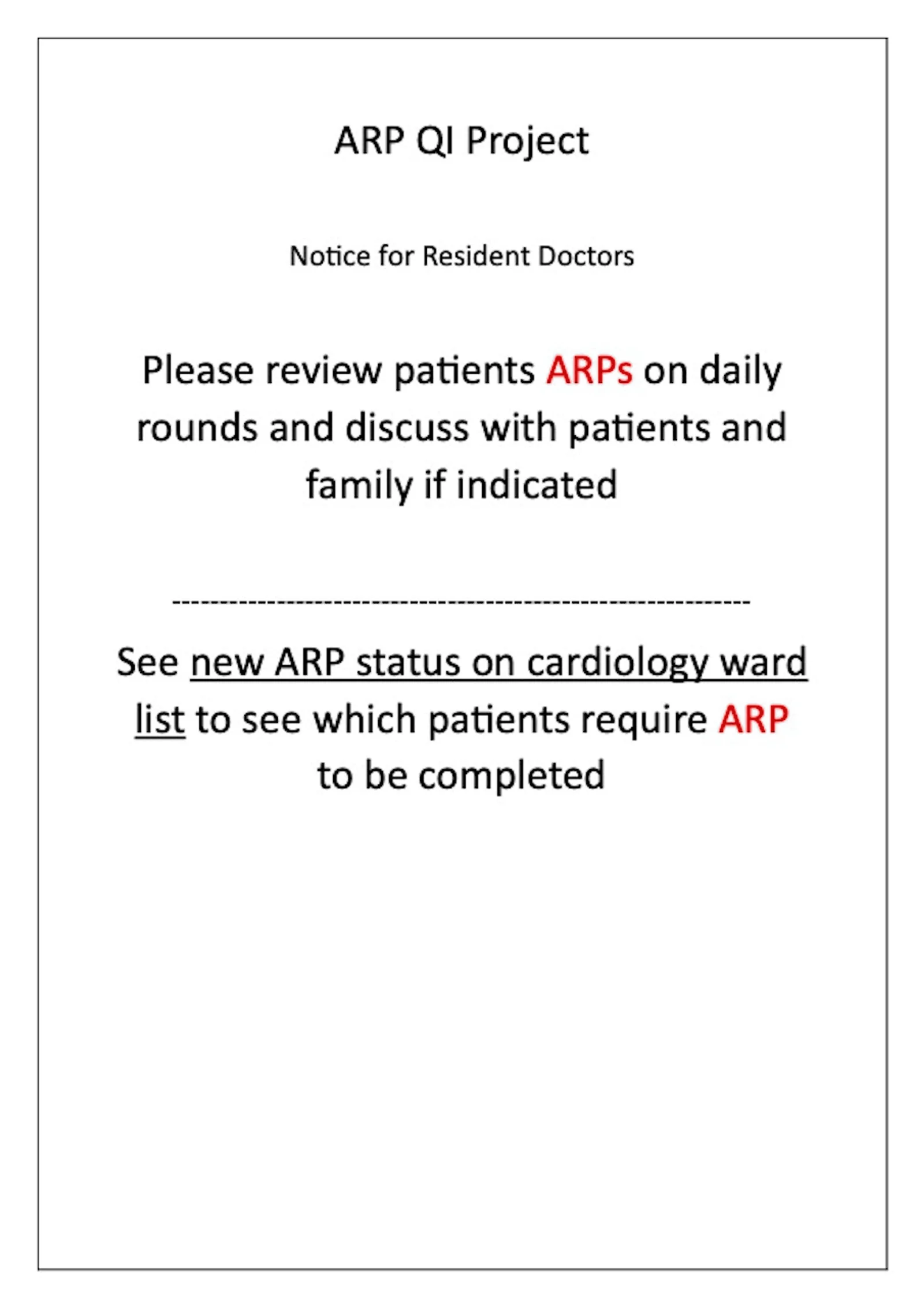
Study poster used during the study, which was placed at clinical workstations ARP QI: Acute Resuscitation Plan Quality Improvement

The second cycle of interventions involved placing visual prompts on the mobile computer workstations of the cardiology team, which are used during ward rounds to access the IEMR, again encouraging the completion of ARPs and discussion with patients and their families.

Ethics

This study was conducted with the knowledge and support of the senior clinicians in the Department of Cardiology and was conducted with verbal consent and in line with the research and ethical guidelines set out in the hospital trust in which the study took place. This study did not use or collect any patient-identifiable information. Therefore, the need for individual patient consent for participation in the study was not required. Given this project’s aim was to alter the behaviour of clinicians and no patient-identifiable information was gathered, IRB approval was not required for this study.

Statistics

The results were assessed for statistical significance using Fisher’s exact test with pairwise comparisons. Results considered significant were defined as having a p-value < 0.05.

## Results

In the initial baseline audit consisting of 25 cardiology inpatients, only 4/25 (16%) had ARPs completed, with the completion rate higher for patients admitted to the CCU than in the cardiology ward at 2/8 (25%) and 2/17 (11.8%), respectively. All the patients in the initial audit who had completed ARPs had an active DNACPR order.

Following the first cycle of interventions, completion rates markedly improved to 10/21 (47.6%, p = 0.027). This improvement was seen across both CCU and the cardiology ward, to 4/7 (57.1%) and 6/14 (42.9%), respectively. Following the second intervention, completion rates improved more modestly, to 12/23 (52.2%), with completion rates of 5/8 (62.5%) in CCU and 7/15 (46.7%) in the cardiology ward, respectively. This was significantly higher than the baseline results (p = 0.014) but did not demonstrate a significant increase from the first cycle (p = 1.000). These results show that the presence of visual aids in clinical spaces increased the completion rate of ARPs.

Each cycle of the project contained separate cohorts of patients with no repeating patients between cycles. For reasons of patient confidentiality, information regarding the exact dates of ARP discussions that took place or the duration of ARPs for individual patients was not collected.

Aggregating post-intervention results across both cycles and analysing them using Fisher’s exact test revealed a statistically significant sustained improvement in completion rate of ARPs compared to baseline (p = 0.019). These results can be seen in Table 1.

**Table 1:** ARP and DNACPR completion rates after each cycle of interventions Table showing the completion rates of ARPs and DNACPRs at baseline and after each cycle of interventions. The numbers in brackets correspond to the number of patients with an active ARP or DNACPR out of the total number of patients in that cohort. This shows the increased engagement of ARPs by clinicians with each cycle of interventions, with improvements seen across all clinical areas. P-values were calculated using Fisher's exact test. ARP: Acute Resuscitation Plan; DNACPR: do not attempt cardiopulmonary resuscitation; CCU: coronary care unit

Audit point during the project	All cardiology inpatients	CCU inpatients	Cardiology ward inpatients	P-value vs baseline (All)	Active DNACPR orders	P-value vs baseline (DNACPR)
Baseline	16% (4/25)	25% (2/8)	11.8% (2/17)	N/A	16% (4/25)	N/A
Cycle 1	47.6% (10/21)	57.1% (4/7)	42.9% (6/14)	p = 0.027	28.6% (6/21)	p = 0.475
Cycle 2	52.2% (12/23)	62.5% (5/8)	46.7% (7/15)	p = 0.014	30.4% (7/23)	p = 0.311

ARP completion was consistently higher in patients admitted to CCU than on the inpatient ward, which may represent patients in this setting having a more complicated or unstable clinical picture, prompting clinicians to consider escalation plans. The completion rates between the CCU and the inpatient ward are demonstrated in Figure 2.

**Figure 2:**
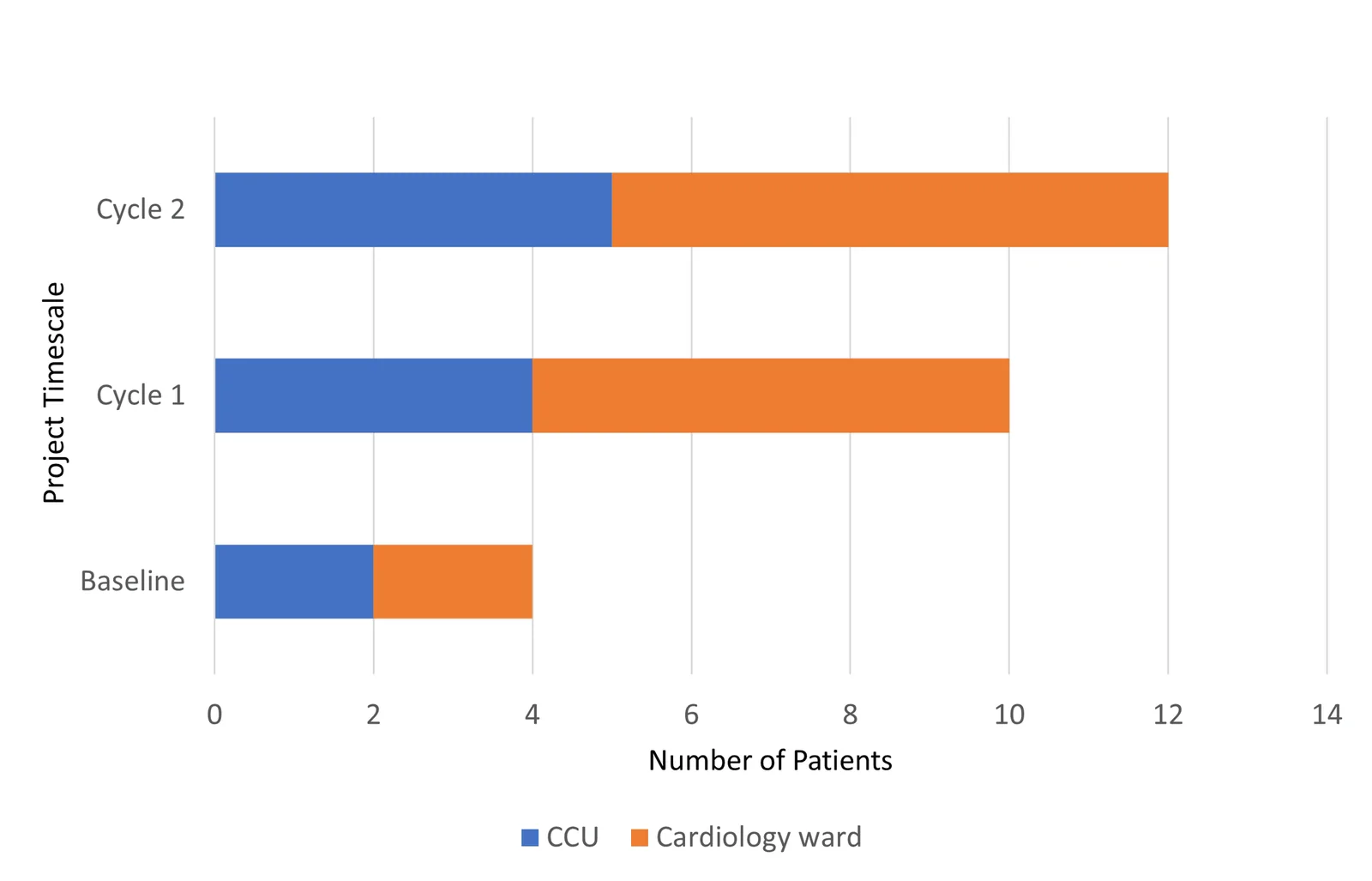
Location of patients with a completed ARP across all cycles of the project Bar chart showing the distribution of completed ARPs between CCU and the inpatient ward across all cycles of the study. The share of completed ARPs grew for patients admitted to the cardiology inpatient ward as the study progressed, reflecting increased engagement with ARPs for the inpatient ward cohort specifically compared to baseline. ARP: Acute Resuscitation Plan; CCU: coronary care unit

The completion rates of DNACPR orders also increased by a smaller amount, to 6/21 (28.6%, p = 0.475) and 7/23 (30.4%, p = 0.311) in the first and second cycles, respectively. On statistical analysis, this small increase in results was not considered a significant rise compared to baseline, suggesting our interventions had no meaningful effect on completion of DNACPRs as part of ARPs (p = 0.450). These results are reflected in Figure 3.

**Figure 3:**
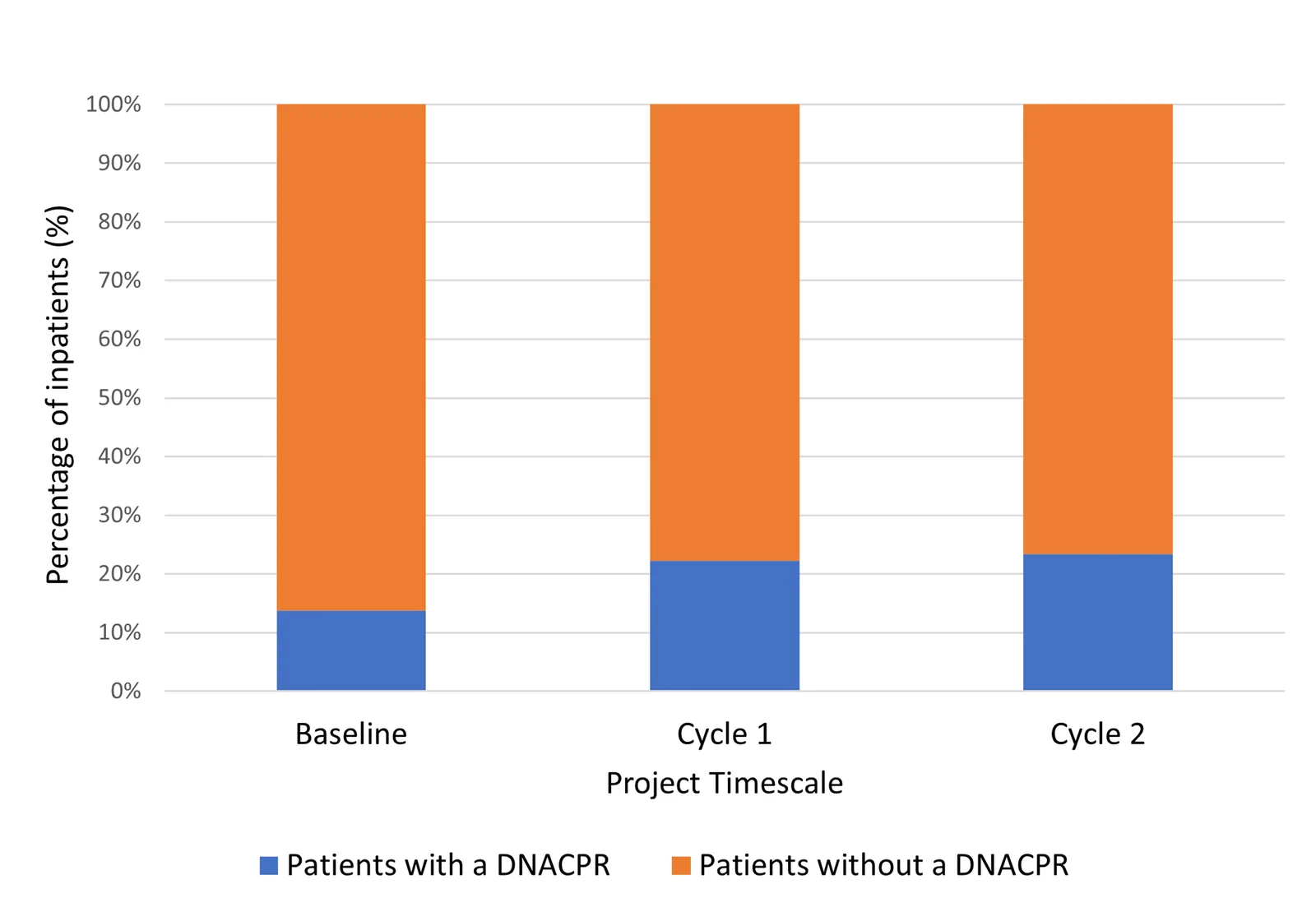
DNACPRs as a percentage in the inpatient cardiology population across all cycles Bar chart showing the proportion of DNACPRs as a percentage of the total cardiology inpatient population. This shows the modest increase in the number of DNACPRs across the project and reflects that most inpatients remained candidates for CPR. DNACPR: do not attempt cardiopulmonary resuscitation; CCU: coronary care unit

## Discussion

This project has shown that the use of visual aids is associated with an increase in ARP completion rates in an inpatient district general cardiology setting over a short timeframe. The results show an increase in engagement from clinicians following the introduction of visual aids to clinical workspaces across all areas related to the Department of Cardiology.

There are several previous small studies that have looked at increasing ARP completion in an inpatient setting [10-16]. These projects were undertaken in several specialties including orthopaedics, respiratory, and geriatric medicine; we were unable to find any reports of projects similar to this in an inpatient cardiology setting [10-16]. Previous studies had varying degrees of success; Quarm et al. did not note any significant rise in ARP completion following their interventions [10], whilst Choo et al. noted significant improvement [11]. There were a variety of interventions used in these studies, including one-off teaching sessions for clinical staff, multidisciplinary team (MDT) discussions, the production of educational materials, and visual aids [10-16]. None of these studies focused purely on the use of permanent visual aids as was described in this study.

The interventions in this study were low-cost, time-efficient, and straightforward to implement across clinical areas, which may allow our interventions to be more easily replicated. The study also intended for visual aids to act as effective prompts, without burdening busy clinicians. In contrast, previous studies have often relied on more resource- and time-intensive interventions, such as developing institutional algorithms, organising formal teaching sessions, or continuously tracking data to produce departmental rankings [13,15,16]. In addition, the use of visual aids within patient-facing spaces aimed to encourage greater engagement at the time of patient interaction, compared to interventions that take place away from clinical areas, such as teaching sessions.

Of the two cycles of interventions in this study, the first had a far greater effect. This may indicate that our second intervention was not as effective as the first. It may also indicate that several of the clinician-related factors were addressed in the first round of interventions, and that the remaining uncompleted ARPs may stem from non-clinician-related factors, meaning a more modest rise was to be expected [17]. Further studies are required to assess whether these initial visual aids were inherently more effective, or if their impact was exaggerated by being introduced in the first round of interventions.

The rate of DNACPR completion rose over the course of the study but was not considered statistically significant. This suggests that our interventions worked well to improve compliance with ARPs but did not impact clinicians' decision-making regarding patients' suitability for CPR. Nonetheless, as seen in previous studies, increasing engagement with ARPs improves overall resuscitation planning and can prevent unnecessary and unwanted interventions to patients, even when CPR is considered appropriate [3].

To further facilitate improvement in ARPs, future projects could focus on more active interventions in addition to the passive interventions described in this project, including raising ARP status at clinical handover and multi-disciplinary team meetings or by working with hospital information technology (IT) departments to create reminders in the IEMR, which have also been shown to increase ARP completion [11,12]. Previous studies have emphasised the importance of early ARP completion [3], so future interventions should aim to target ARP completion at admission rather than on ward rounds, as in this study.

There were several limitations to this project. Firstly, it was conducted in a single cardiology department, so the number of individuals impacted by the interventions in this report is relatively small. The monitoring period of three weeks was also relatively short, meaning the long-term outcomes of the interventions could not be fully assessed. In the first cycle, two interventions were introduced simultaneously, meaning the impact of these interventions separately could not be assessed. A further limitation of the study, being conducted in a single cardiology unit, meant we were unable to have a concurrent control group of clinicians and patients not exposed to visual aids. Given the small sample size in this study, for reasons of patient confidentiality, we were unable to assess if any individuals may have inappropriately received resuscitation.

## Conclusions

This project has demonstrated that the use of visual aids was associated with an increase in ARP completion rates in an inpatient district general cardiology department over the course of three weeks, although there was no significant effect on the number of DNACPR forms completed. These findings show that the use of visual aids was associated with increased engagement with ARPs and could be used in a more widespread setting to encourage discussions with patients and their families regarding resuscitation planning. To fully assess the impact of visual aids on ARP completion rates, larger-scale projects with an increased sample size and monitoring over a longer period are required.
